# Microbes in porous environments: from active interactions to emergent feedback

**DOI:** 10.1007/s12551-024-01185-7

**Published:** 2024-04-19

**Authors:** Chenyu Jin, Anupam Sengupta

**Affiliations:** 1 Physics of Living Matter Group, Department of Physics and Materials Science, University of Luxembourg, 162 A, Avenue de la Faïencerie, Luxembourg City, L-1511 Luxembourg; 2 Institute for Advanced Studies, University of Luxembourg, 2 Avenue de l’Université, Esch-sur-Alzette, L-4365 Luxembourg

**Keywords:** Microbial active matter, Porous environment, Dispersal, Colonization, Feedback

## Abstract

Microbes thrive in diverse porous environments—from soil and riverbeds to human lungs and cancer tissues—spanning multiple scales and conditions. Short- to long-term fluctuations in local factors induce spatio-temporal heterogeneities, often leading to physiologically stressful settings. How microbes respond and adapt to such biophysical constraints is an active field of research where considerable insight has been gained over the last decades. With a focus on bacteria, here we review recent advances in self-organization and dispersal in inorganic and organic porous settings, highlighting the role of active interactions and feedback that mediates microbial survival and fitness. We discuss open questions and opportunities for using integrative approaches to advance our understanding of the biophysical strategies which microbes employ at various scales to make porous settings habitable.

## Introduction

Microorganisms including bacteria, archaea, fungi, and diverse protists, have successfully inhabited the biosphere, pervading several kilometers into the atmosphere, the oceans, the seabeds, and the depths of subsurface rocks. With the total number of prokaryotes (bacteria and archaea) on Earth estimated to be about $1.2 \times 10^{30}$ cells (Flemming and Wuertz (2019)), they account for nearly $15\%$ of the net biomass on Earth, despite being orders of magnitude smaller size than those of the eukaryotic cells (Bar-On et al. (2018); Philippot et al. (2023)). Four of the “big five” habitats where the majority of microbes are found: soil, oceans, deep continental subsurface, deep oceanic subsurface, and the upper oceanic sediments, are of a porous nature and host around $90\%$ of all microorganisms (Ebrahimi and Or (2014); Dang and Lovell (2016); Flemming and Wuertz (2019)). In addition, microbes often inhabit living and decaying plant and animal bodies, exploiting symbiotic or pathogenic relationships. Such organic substrates, including mucus linings (Kirch et al. (2012); Bansil et al. (2013); Bansil and Turner (2018); Wu et al. (2023)), food materials (Aminifar et al. (2010); Ranjbaran et al. (2021); Dadmohammadi and Datta (2022)), and flocculated suspended sediments (flocs) including marine snow (Kiørboe et al. (2002); Nguyen et al. (2017); Lawrence et al. (2023); Borer et al. (2023)), offer dynamic porous environments where microstructure and local viscoelasticity mediate microbial dispersal, locomotion and colonization over time.

Despite their tiny size, microbes are evolutionarily well equipped to disperse widely, relying on a range of passive and active mechanisms. Taking *Bacillus subtilis* as an example, this common soil bacterium is only a few microns in size but manages to colonize soil across all continents (Philippot et al. (2023)) and in underground water columns several kilometers deep into the soil (Kondakova et al. (2013)). Microbial communities inhabiting porous subsurface environments have received particular attention in recent years due to their implications in hydrogen (H_2_) storage. H_2_, an important potential replacement of fossil fuels, has necessitated the search for new storage facilities in the form of depleted gas fields and aquifers (Heinemann et al. (2021); Muhammed et al. (2022)). However, our understanding of the behavior and impacts of stored H_2_ within such porous settings, particularly in the context of a diverse microbial ecosystem thriving in these environments, is at a nascent stage (Thaysen et al. (2021); Liu et al. (2023a)). Due to the anoxic conditions, subsurface microbial communities may utilize H_2_ as an electron donor, producing various gaseous by-products including hydrogen sulfide and methane. Biological activity and microbial growth over time lead to the formation of biofilms, with subsequent clogging of the subsurface pores and a significant increase in pressures required for gas injection (Eddaoui et al. (2021)). In addition, gaseous metabolites generated by microbes can alter the pore-scale wettability, while interaction with water molecules can trigger the formation of acidic compounds, leading to behavioral and physiological impacts on the microbial communities (Liu et al. (2019); Santha et al. (2019); Pan et al. (2022)).

**Fig. 1:**
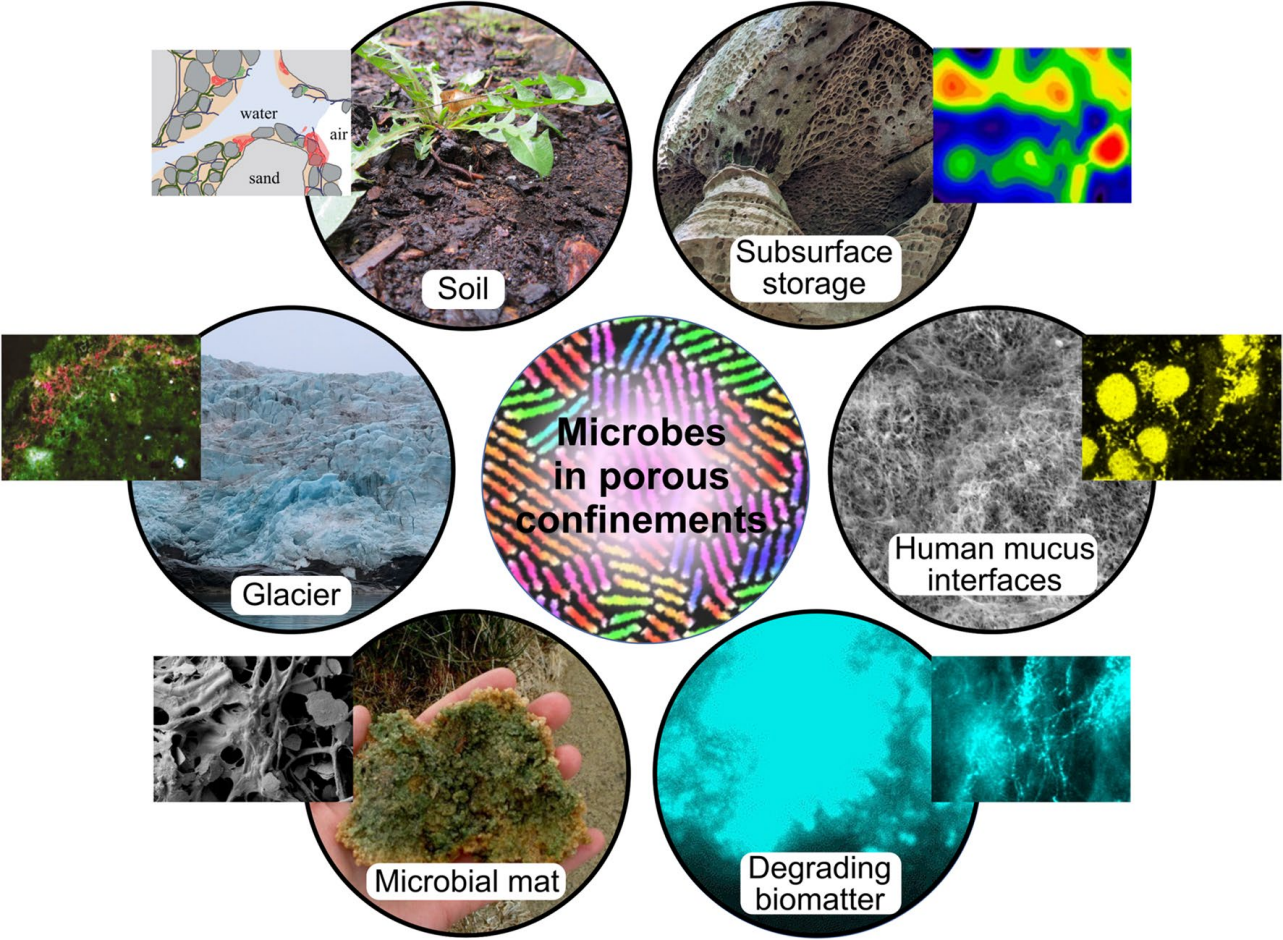
Microbes inhabiting porous environments. Clockwise from top: Soil is a packed, organo-mineral complex. Side panel shows the microscopic inner structure of soil. Subsurface main panel shows honeycomb-patterned sandstone in Mullerthal region of the Grand Duchy of Luxembourg. Subsurface inner structure shows contour plots of hydrogen gas saturation in the pore network during bacterial growth, adapted from Liu et al. (2023b), available under the terms of CC BY 4.0. Human mucus found in the gut, lungs and urinary tract offer habitats to diverse bacterial species. Side panel shows the fluorescence in situ hybridization (FISH) image of biofilms formed by native salivary communities over 48 h, adapted from Wu et al. (2023), CC BY 4.0. Degrading planktonic matter represents a hierarchical fibrous structure (shown in cyan), acting as a chemo-attractant for motile bacterial species. A benthic microbial mat found in the wetland. The side panel shows the SEM image of the internal structure of the microbial mat, and mineral particles associated with filamentous cyanobacteria. Panel adapted from Power et al. (2007), CC BY 2.0. Glacier main panel is taken at Billefjorden, Norway by Xinran O. Zhao. Side panel shows the inner structure of a cryoconite aggregates, the red hue indicates cynobacteria (viewed under UV), adapted with permission from Reference Hodson et al. (2010), copyright 2010 Cambridge University Press

Microbes disperse through porous confinements by both passive pathways (Conrad and Poling-Skutvik (2018); Rhodeland et al. (2020)) and active biophysical mechanisms (Tecon and Or (2017); Scheidweiler et al. (2020); Balseiro-Romero et al. (2022)). Passive pathways include convective transport due to fluid flow, attachment and subsequent transport by soil particles or hitchhiking larger organisms inhabiting pore spaces, such as worms, nematodes, and insects. Active dispersal mechanisms, typically under hydrated conditions, may involve surface-mediated movement by swarming, twitching, gliding, or sliding, often mediated by physico-chemical gradients that exist or develop locally. Due to the microscale body length of bacteria, their active dispersal is spatially limited (Wisnoski and Lennon (2023); Wu et al. (2023)). To date, most studies of microbial ecology in porous environments, particularly in the context of biogeography, have primarily considered passive dispersal. However, several recent papers have shown how active dispersal, aided by the dynamic transformation of microbial life forms between sessile and planktonic states could play a critical role in microbial colonization, interactions and community-scale resilience in diverse porous confinements (McDougald et al. (2012); Smith et al. (2018); Coyte et al. (2017); Taubert et al. (2018); Abiriga et al. (2022); Soares et al. (2023)).

Microbes occupy diverse porous settings (Fig. 1), each exhibiting distinct biophysical properties which mediate microbial dispersal and locomotion in different ways. For instance, the food that we eat is a spatially structured complex soft material that has been extensively investigated in the framework of porous media (Ranjbaran et al. (2021); Dadmohammadi and Datta (2022)). Soil, an archetypical example of a porous environment, contains about one-quarter of total prokaryotic cells concentrated in a small volume (Flemming and Wuertz (2019)), hosting approximately $3 \times 10^{29}$ prokaryotic cells. On the one hand, the production of extracellular polymeric substances often leads to bioclogging of the porous networks, on the other hand, they also enable water retention keeping soils alive and fertile (Philippot et al. (2023); Volk et al. (2016); Rabbi et al. (2020)). The intrinsic structural heterogeneity, offered by the packed, organo-mineral complex and hierarchical porosity, provides suitable niches for different microbial species (Philippot et al. (2023)). Microaggregates arising from sand, silt, and clay are relatively labile being loosely held together by polysaccharides, root, and fungal hyphae. Overall, the soil body is susceptible to various physico-chemical changes over daily to seasonal timescales, resulting in a heterogeneous and fluctuating microbial distribution (Tecon and Or (2017)).

Another microbially relevant porous setting is the subsurface rocks, covering the Earth’s crust and deep-sea sediments. Despite the extreme environmental conditions, characterized by lack of light, lack of oxygen, and high pressures and temperatures, they harbor active microbial communities (Escudero and Amils (2023)). The presence of water in such extreme environments (made possible by fractured and highly porous rocks) promotes the formation of multi-species biofilm communities even at depths of hundreds of meters below the surface (Escudero et al. (2018)). Subsurface rocks, together with aquifers, recently attracted attention for their potential use as H_2_ reservoirs (Heinemann et al. (2021); Tarkowski and Uliasz-Misiak (2022)). Yet systematic studies are yet to pick up, which could help to understand the interactions and feedback between H_2_ and the subsurface microbial communities in porous settings. H_2_-mediated metabolic processes may alter the pore-scale properties (Liu et al. (2023a)), with implications on the suitability of subsurface rocks as reservoirs (Heinemann et al. (2021); Thaysen et al. (2021)).

Emerging climatic shifts have resulted in large-scale thawing of glaciers, leading to the formation of slushy, saturated, porous matrix rich with dicer microbial communities (Stevens et al. (2022)). Characterized by a shallow ($<2\,\textrm{m}$) “weathering crust” with the cryoconite (“cold dust”) holes piercing the continuous ice surface (Cook et al. (2016)), these active microbial hotspots harbor millimeter-size aggregates of microbes and silt, held together by the ice crystals (Hodson et al. (2010)).

Finally, diverse biological systems represent active, porous environments spanning orders of length scales. These include bones, which are rigid porous structures filled with cells, extracellular matrix, and biological fluids; the circulatory system comprising hierarchical porous capillaries for blood, lymph, and gas transport; and microbe-rich mucus linings which are deformable cross-linked polymer networks filled with viscoelastic fluids, with average pore sizes in the range of hundreds of nanometers (Bansil et al. (2013); Bansil and Turner (2018).) Pathogenic bacteria are often found to swim in the lungs of patients suffering from cystic fibrosis (Zlosnik et al. (2014)), while T-cells are versatile in their ability to navigate tissues such as liver, lymph nodes, and lungs (Rajakaruna et al. (2022)). Furthermore, modern medical technologies and biomedical diagnostics use active navigation strategies for disease detection and drug delivery (Gao et al. (2021); Zhang et al. (2021); Wu et al. (2018); Wan et al. (2020)). Finally, the bacterial biofilm itself represents a living porous system. Embedded within an extracellular matrix composed of polymers and proteins, they act as active porous structures that evolve over time (You et al. (2018, 2019); Carpio et al. (2019)), depending on local physical factors such as substrate curvature (Schamberger et al. (2023); Langeslay and Juarez (2023)), structural anisotropy (Vallespir Lowery and Ursell (2019)), and substrate stiffness (Lin et al. (2020); Sengupta (2020); Asp et al. (2022)). Interestingly, the porosity of the biofilm influences the growth rate of individual cells: cells occupying loosely packed regions of a colony show a higher rate of elongation compared to cells positioned in tightly packed regions of the biofilm (Wittmann et al. (2023)).

In this review, we present key advances in our understanding of active microbial strategies within diverse porous environments (covering the last 15 years), with an overarching aim to identify universal local properties and mechanisms which microbes put to use across different microstructural and physico-chemical constraints. This review is organized as follows: After the short introduction to microbe-inhabited porous environments (“Introduction’’ section), we summarize the associated biophysical conditions and length scales (“Porosity and length scales of microbe-inhabited porous environments’’ section), and then move to the “ Active microbes in dynamic porous confinements’’ section, where we present recent literature on active strategies of bacterial dispersal, spanning diverse microbial families including archaea, algae, and fungi. In the “ Summary and perspectives’’ section, we offer future opportunities and key open questions as well as challenges in the field of microbial organization in porous environments.

**Fig. 2:**
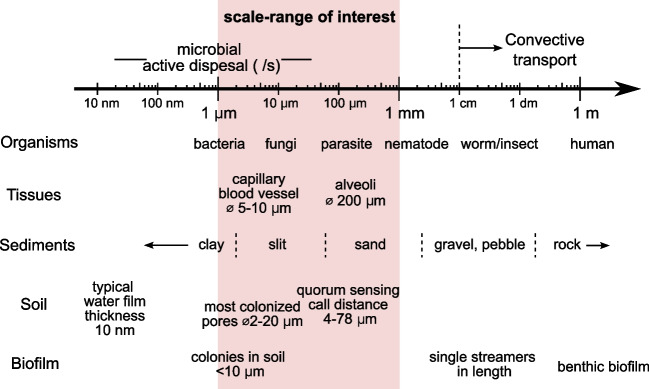
Characteristic length scales in microorganisms and their porous habitats. The shaded red region matches the typical microbial length scales and represents the range of dimension covered in this review. Most of the numbers in the figure is also presented in the main text and from the following works of Dexter (1988); Ranjard and Richaume (2001); Grundmann (2004); Raynaud and Nunan (2014); Gantner et al. (2006); Battin et al. (2016).

## Porosity and length scales of microbe-inhabited porous environments

Porosity, a key metric which characterizes porous environments, is a measure of the relative void space in a given volume. Formally, this is defined as the ratio between the pore volume and the total volume (also referred to as the bulk volume), expressed either as a fraction or in percentage (Giao and Nguyen (2022)). For further definitions of porous environments and their mathematical descriptions (for example, in terms of the hydraulic conductivity or network analyses), we would request readers to refer to the classic work of Vogel (2000). Specifically, in the context of soil microbial ecology and biofilms in confined flows, detailed literature covering relevant porosity values, as well as the biophysical conditions (temperature and local humidity) can be found in the works of Tecon and Or (2017), and Conrad and Poling-Skutvik (2018). Building on these existing studies, here we focus on the active dispersal strategies of microbes in diverse porous media settings.

Natural porous media are hierarchical in structure, with corresponding length scales spanning several orders of magnitude from sub-micron to millimeter length scales. Some typical length scales are summarized in Fig. 2. The small size of microorganisms makes them well-suited to live inside the pores. In the soil, quantitative studies show that the majority of bacteria are found in the micropores within soil aggregates (Han et al. (2021)). Depending on the soil texture, the maximum diameter of the pores most frequently colonized by bacteria, has been estimated to be between 2.5 and $9 \,\mu \textrm{m}$, while no bacteria have been observed in pores smaller than $0.8 \,\mu \textrm{m}$ in diameter (Ranjard and Richaume (2001)). The typical size of a *Bacillus subtilis* bacterium is 4–$5 \,\mathrm {\mu m}$ in length and 0.75–$1\,\mathrm {\mu m}$ in diameter. Experiments with microfluidic devices also show that the narrowest slit through which bacteria can pass is $0.75 \,\mu \textrm{m}$ for *B. subtilis* and $0.4 \,\mu \textrm{m}$ for *E. coli* (1–$2 \,\mathrm {\mu m}$ in length and 0.25–$1\,\mathrm {\mu m}$ in diameter) (Männik et al. (2009)). However, it still remains to be confirmed whether micropores act as hotspots for active microbes, or provide protective habitats under unfavorable conditions, or simply are passive dead-ends where cells are trapped (Totsche et al. (2018)). Interestingly, in general, microbial colonies inside soil are tiny compared to lab-grown biofilms. For instance, *B. subtilis*, which forms $\approx 10\,\textrm{cm}$ large biofilms on nutrient-rich agarose culture plate, usually appears as small colonies of two to five cells (Grundmann (2004)).

Motile microbes can swim at a speed of about one body length per second. The persistent swimming speed of *Bacillus subtilis* can reach $20\,\mu$m/s (Najafi et al. (2019)), while the parasite *Trypanosoma brucei* (8 to $50 \,\mathrm {\mu m}$ long) swims at a average speed of $12\,\mathrm {\mu m/s}$ (Bargul et al. (2016)). Such a speed suggests that the active dispersal of microbes occurs at the pore scale as well as at scale of microbial body size, thus plays a key role in the accessing and colonization of porous environments. Both velocity and diffusion limit the range of cell-cell interactions. Gantner et al studied the quorum sensing in the rhizosphere, and found that the effective “call distance” was between 4 and $78 \,\mathrm {\mu m}$ depending on the environment (Gantner et al. (2006)). Raynaud and Nunan analyzed microbial colonization patterns based on microscopic images of several hundred thin sections of soil. They observed an average cell-to-cell distance of about $12.5 \,\mathrm {\mu m}$ in the soil matrix (Raynaud and Nunan (2014)).

**Fig. 3:**
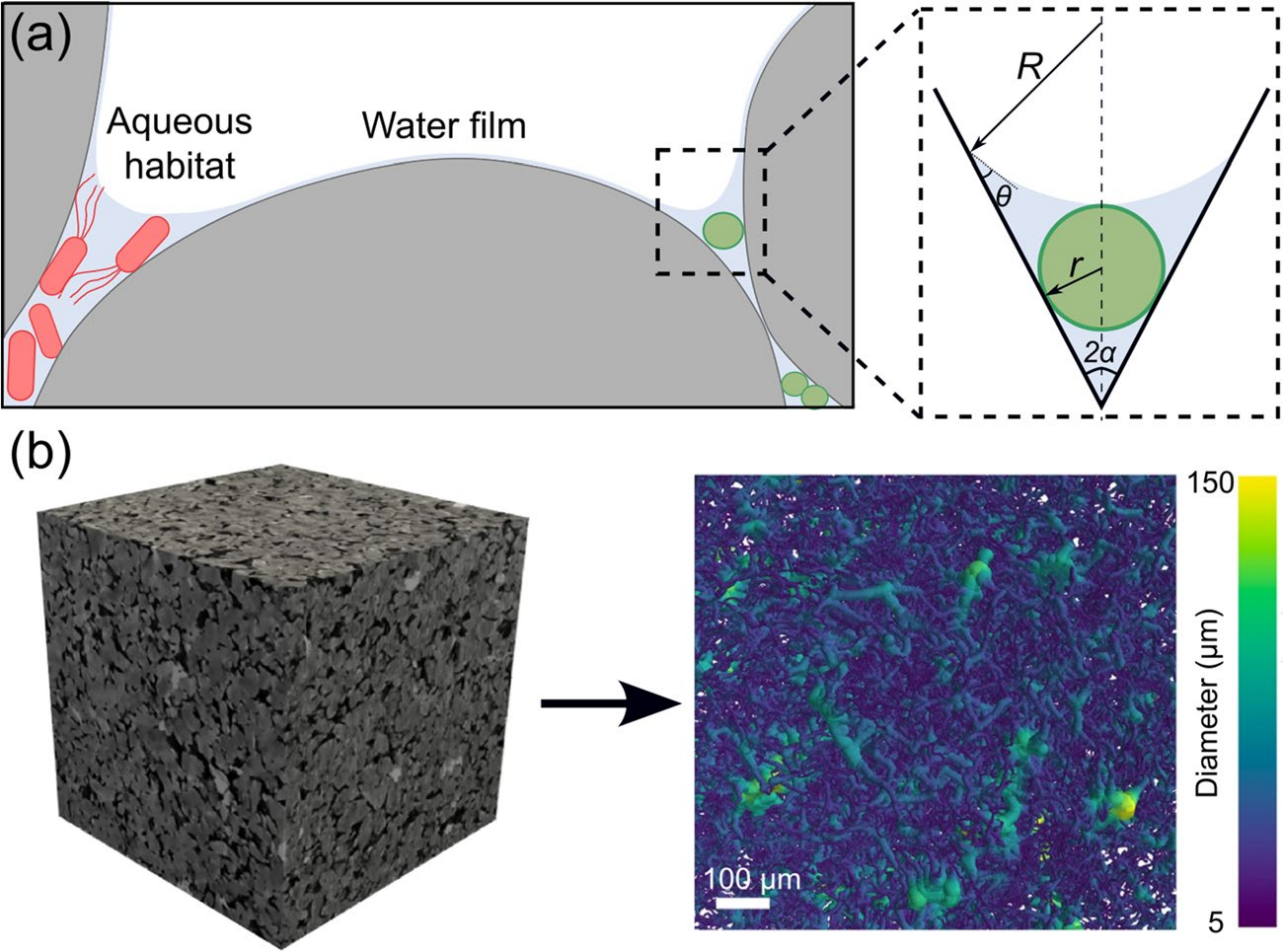
Porous environments impose a strong confinement on microorganisms. **a** Schematic representation of water distribution in a porous unsaturated environment, e.g., soil. The size of the confined aqueous zone, shown here in the corner, is determined by the contact angle and the angle between the solid surfaces. Depending on the size of the aqueous zone, the resident microbe could be completely or partially entrapped. **b** Pore network representation of a rock sample ($2.25\, \textrm{mm}$ side length) derived from an X-ray microtomography, color-coded by local diameter (Neumann et al. (2021)), CC BY 4.0

## Active microbes in dynamic porous confinements

Microbes growing in porous environments are active systems where an interplay of confinement-induced physical and chemical constraints determine microbial behavior, physiology, and self-organization (Araújo et al. (2023)). Porous surfaces can modulate the transport of microroganisms through steric, electrostatic, chemical, and/or hydrodynamic interactions, each with a characteristic interaction range (Conrad and Poling-Skutvik (2018)). Furthermore, transport processes in porous environments are mediated by the pore-scale network and topology (Hazas et al. (2022)). While random networks in most natural porous media are not perfectly connected (as shown in Fig. 3b), the connectivity decreases further as the hierarchical length scale increases. This results in the formation of “dead ends” that can only be reached by diffusion or active dispersal of microorganisms.

It should also be noted that most parts of the soil and subsurface are unsaturated with most of the water accumulates in the nooks and corners, as well as the micro- and mesopores. It is the network of these aqueous elements, rather than the entire void, that limits both nutrient diffusion and microbial movement (Fig. 3a) (Tecon and Or (2016)). Specifically, Or et al estimated that in a typical soil with moderate moisture (soil matric potential $-30\, \textrm{kPa}$), at a $60^o$ corner formed by two mineral surfaces, the aqueous element is just enough to submerge a single bacterium with a radius of $\approx 1\, \mathrm {\mu m}$ (Or et al. (2007)), but will limit the flagellar beating and hence the motility of microorganisms (Dechesne et al. (2010); Tecon and Or (2016)). The thin water film on a smooth mineral grain surface in such a soil ($<10\,\textrm{nm}$) will also limit the diffusion of chemicals (Or et al. (2007)). Both can lead to critical metabolic consequences for the microbes (Rivkina et al. (2000)). Below is a brief overview of the different motility mechanisms that microbes use to navigate porous environments. More details on bacterial motility mechanisms can be found in Wadhwa and Berg (2022); Thormann et al. (2022).

### Microbial motility mechanisms

#### Swimming

Many microorganisms switch from a biofilm to a planktonic life form thanks to the flagella, which enables the cells to swim in aqueous environments after detachment from the mother colonies. Flagella are helical appendages, typically 5 to $10 \,\mathrm {\mu m}$ long and $20 \,\textrm{nm}$ in diameter, that are driven by molecular motors at the expense of energy units (ATP) to propel the cell body forward (Yonekura et al. (2003); Grognot and Taute (2021a)). Although metabolically costly (Schavemaker and Lynch (2022)), swimming not only allows microorganisms to navigate but also plays an important role in microbial colonization under flow (discussed in the next section).

Over long timescales, bacterial swimming can be modelled as diffusion, involving straight-run events, interspaced with reorientation events that allow cells to navigate and sample their environment. There are several reorientation mechanisms, the most thoroughly studied being “run-and-tumble”: by changing the rotational state of the flagellar filament, the bacterial body alternates the state between “run” (approximately 1s) and “tumble” (approximately 0.1s), exhibiting a random walk on a large time scale (Lauga (2016)). For a long time, the run-time was considered to be Poisson distributed, but a recent study shows that there is a large behavioral variability due to the fluctuation of the signal protein (Figueroa-Morales et al. (2020)). Under certain assumptions, the coarse-grained fluctuating hydrodynamics of interacting ABPs and run-tumble particles can be mapped onto each other and are therefore strictly equivalent (Cates and Tailleur (2013)).

Bacteria are able to move towards nutrient, oxygen, and quorum sensing signals, and away from predator signals. Such directional movement guided by an external chemical field is called chemotaxis (Keegstra et al. (2022)). By moving around, bacteria sample the chemical concentration in the environment, and then, based on very limited information, bias their random walk along / against the chemical gradient by reducing tumbling (Mattingly et al. (2021)). Confinement, including crowding due to high cell density, can affect the sampling process and hence the chemotaxis (Grognot and Taute (2021b); Colin et al. (2019)). Chemotactic cells, even with some degree of phenotypic variability, can self-organize into travelling bands (Fu et al. (2018); Bhattacharjee et al. (2022)). Such travelling bands can sweep rapidly across complex geometries, e.g., porous media (Adler (1966); Budrene and Berg (1995); Bhattacharjee et al. (2021)), and are therefore used by microbes as an efficient active dispersal strategy (Cremer et al. (2019); Ni et al. (2020)).

#### Swarming

Swarming is a second form of flagella-mediated motility, typically observed when planktonic microbes grow in nutrient-rich environments. Under these conditions, cells become multinucleated, elongate, grow large numbers of flagella (hyperflagellation), and secret bio-surfactants to march across surfaces in coordinated rafts (Kearns (2010)). Tumble mode is suppressed during swarming (Partridge et al. (2020)). The water content of the medium is a key determinant of swarming: while too much water promotes swimming, too little water inhibits swarming. Wetting agents, such as secreted biosurfactants and lipopolysaccharide (LPS), often aid swarming motion by maintaining an optimal moisture content of the solid surface. To the best of our knowledge, there is currently no data on the optimal water content and film thickness required for swarming. Reports by Zhang et al. have estimated the liquid film to be approximately 10 times thicker at the edge of the swarm than on the virgin agar (Zhang et al. (2010)), while Wang et al. have found that spraying water (approximately $3\,\mu \textrm{L}$ per $\mathrm {cm^2}$ of agar surface) stimulated cells to swarm normally (Wang et al. (2005)). Although swarming has not been demonstrated in natural environments, a similar form of collective movement of *B. subtilis* towards plant roots has recently been observed in transparent soil (Engelhardt et al. (2022)). This collective movement may be a mechanism to enhance the colonization of the plant hosts.

#### Twitching

Twitching is the bacterial surface movement powered by extension and retraction of type IV pili. The pili extend, attach to the surface and retract. The cell body is slowly pulled forward with a twitchy appearance (Mattick (2002); Craig et al. (2019)). Twitching motility is exhibited by a wide range of bacteria, many of which are pathogenic. It has been observed on both organic and inorganic surfaces, including agar gels, epithelial cells, plastics, glass, and metals (Merz et al. (2000); Mattick (2002)). This form of motility requires a moist surface but not bulk water. Twitching cells share EPS and move in groups to a new colonization site in low water environments at a speed of around $1 \, \mathrm {\mu m/s}$ (Merz et al. (2000); Zhao et al. (2013); Oliveira et al. (2016)). The overall dynamics of twitching is also Brownian, possibly arising due to a “tug-of-war” between the pili located at different points on the cell body, similar to the cargo transported by molecular motors on cytoskeletal filaments (Marathe et al. (2014)). Twitching cells can exhibit chemotaxis (Oliveira et al. (2016)). Twitching mobility also allows bacteria to move against the current and spread upstream (discussed in the next section).

#### Gliding

The term “gliding” generally refers to non-flagellar active surface motions. These are smooth and continuous, and are observed in three major groups of bacteria, the myxobacteria (0.025 to $0.1 \,\mathrm {\mu m/s}$), the cyanobacteria (velocities approaching $10 \,\mathrm {\mu m/s}$), and the Cytophaga-Flavobacterium group (2 to $4 \,\mathrm {\mu m/s}$) (Pfreundt et al. (2023); Faluweki et al. (2023)). Several mechanisms have been proposed to explain gliding motility, including propulsion by contraction of type IV pili (social gliding), transport of macromolecules such as polysaccharides and movement of outer membrane components by protein complexes in the cytoplasmic membrane (adventurous gliding). However, a clear mechanistic picture of how gliding motility is achieved remains to be established.

#### Growth-induced sliding

Sliding is driven by the mechanical force that dividing cells exert on their neighbors, and facilitated by bacterial secretions that help to reduce friction and promote sliding. It is not an active form of movement but is likely to play an important role in bacterial surface colonization, particularly during the early stages of biofilm development when the rate of colony expansion (due to sliding) is proportional to the colony size (Dell’Arciprete et al. (2018); You et al. (2018); Dhar et al. (2022)). More recently, the dispersal of individual cells within a growing colony has been linked to their genealogical separation from the mother cell, revealing the genealogical organization of bacterial cells in a growing colony (Rani and Sengupta (2023)). Growth experiments in natural and artificial confinements have shown that the biomechanical forces generated in the colony are large enough to alter the growth rates of cells in densely packed regions (Wittmann et al. (2023)), and even to squeeze them through slits which are narrower than the cell body (Männik et al. (2009)), suggesting a relevant mechanism by which microbes could penetrate narrow necks to colonize porous confinements.

### Microbial motility across length scales

Despite their small size, microorganisms can move over multiple length scales, and use a variety of motility mechanisms, individually or collectively. The swimming speed of bacteria is quite fast: *E.coli* can swim at about $25 \,\mathrm {\mu m/s}$, or about 20 body lengths per second. However, they often tumble to change direction, which results in a lower speed of propagation. When tumbling is suppressed, they are significantly faster: Cremer et al reported that *E. coli* can move about $30\,\textrm{mm}$ by collective swimming driven by chemotaxis (Cremer et al. (2019)). Swarming cells are even faster, *B. subtilis* can travel $30\,\textrm{mm}$ in just 2.5 h (Kearns (2010)). Interestingly, when *E.coli* moves upstream in a tube, a few pioneer cells reach the distance of $13\,\textrm{mm}$ in about 13 min (Figueroa-Morales et al. (2020)). This is about $17\,\mathrm {\mu m/s}$ against a Poiseuille flow with a maximum velocity of $80\,\mathrm {\mu m/s}$.

Bacteria can also travel centimetres by non-flagellar motions such as twitching, gliding, and growth-induced sliding. However, this takes hours or even days. Twitching *P. aeruginosa* has been observed to move more than $0.7\,\textrm{mm}$ against a flow (velocity 2 to $20\,\mathrm {mm/s}$) in 15 h (Siryaporn et al. (2015)). Different strains of *B. subtilis* on different substrates grown on different substrates have colony fronts ranging from very slow (about $0.05 \,\mathrm {mm/h}$ or $0.016 \,\mathrm {\mu m/s}$) (Grau et al. (2015)) to moderately fast ($5 \,\mathrm {mm/h}$ or $1.6 \,\mathrm {\mu m/s}$) (Kinsinger et al. (2003)).

It is worth noting that a high speed of individuals does not necessarily lead to the high speed of collective migration: At high density, cells are organized into an active nematic structure by steric interactions. If individual cells move too fast, they can become trapped in vortices (local defects). In this case, a lower individual speed actually results in a higher overall migration speed. Meacock et al. have found that this is indeed the strategy used by the wild type *P. aeruginosa* twitching on surface (Meacock et al. (2021)).

### Active colonization strategies in porous confinements

Before they can colonize a site, passively transported microbes have to settle. For example, they must break out of the convective flow fields and attach to a suitable surface (Wheeler et al. (2019)). Their retention is determined by several factors including cell size and shape, motility, and surface properties (Zheng et al. (2021)). In particular, cell motility is generally thought to increase retention in porous media (Liu et al. (2011); Creppy et al. (2019)). However, the precise mechanisms leading to such observations remain to be elucidated, particularly in the context of interactions and biophysical feedback at the cellular and pore scales.

In recent years, the development of microfluidic techniques has enabled quantitative studies of microbial ecology (Rusconi et al. (2014a)). Microfluidic techniques offer flexible and modular built-in structures that mimic the features within porous media, such as confinement, structured geometry, semi-permeable chamber, and controlled flow. Another advantage of microfluidics is its compatibility with optical imaging techniques, which can easily achieve micron-scale resolution at a reasonable cost. Inside a microfluidic chamber, we could use micro-PIV (particle image velocimetry) to measure the flow profile, track the movement of individual bacteria, and identify the orientation of the cell body and even the state of the flagella. With such a wealth of detailed information and mathematical models, researchers are able to uncover more mechanisms. A better understanding of microscale mechanisms leads to significant progress in understanding and modelling of bacteria transport and dispersal at the macroscale. The following is an overview of the processes relevant to microbial dispersion and transport in porous media

**Fig. 4:**
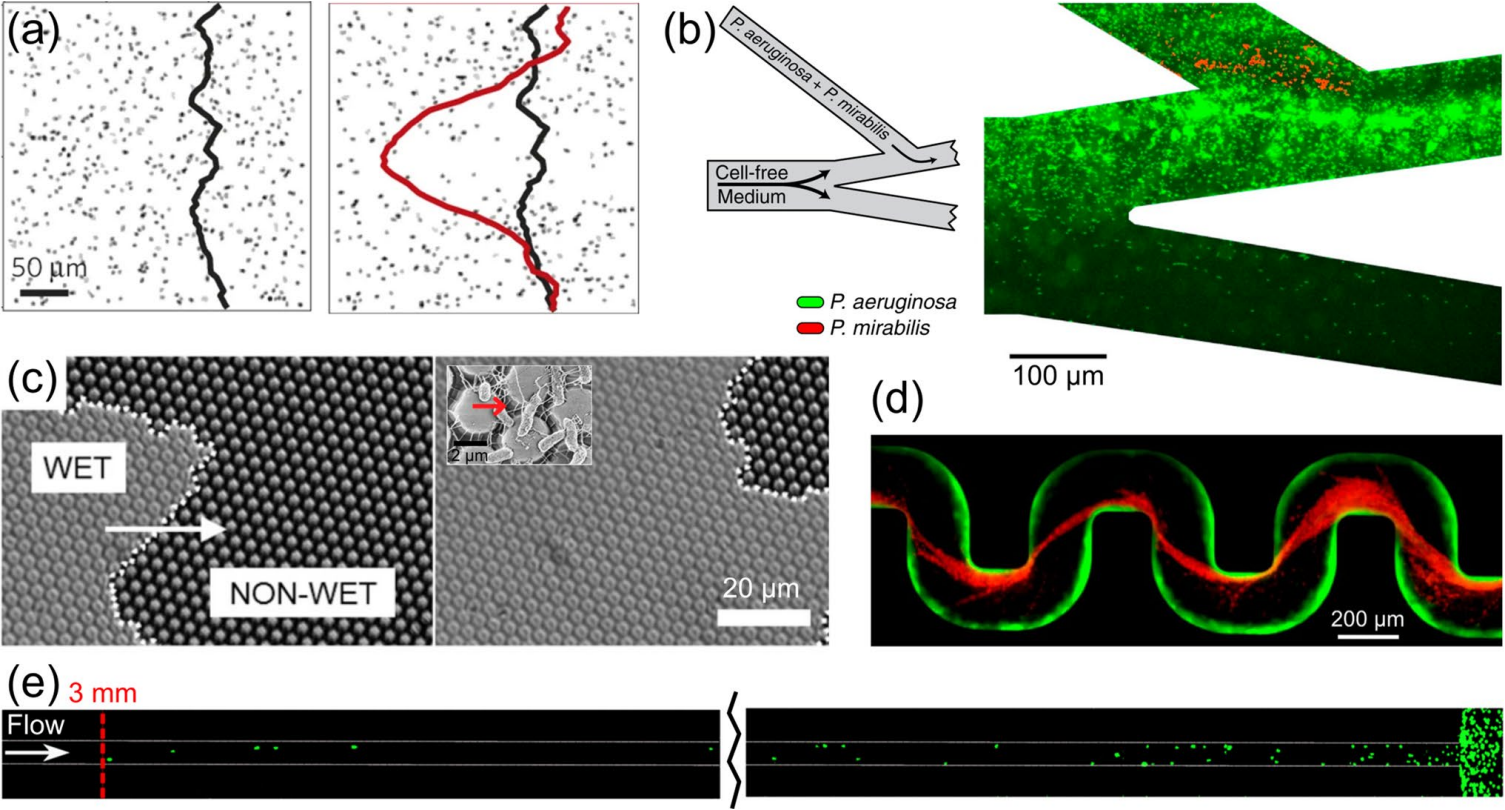
Microbial navigation strategies under confined flows. **a** Shear-induced active retention at surfaces (reproduced with permission from Rusconi et al. (2014b), copyright 2014 Macmillan Publishers Limited). **b** *P. aeruginosa* (green) are able to twitch against flow, hence out-compete *P. mirabilis* in colonizing vasculature-like flow networks (reproduced with permission from Siryaporn et al. (2015), copyright 2015 Elsevier Ltd). **c** Beating of *E. coli* flagella changes the wetting states of the surface (reproduced with permission from Friedlander et al. (2013), copyright 2013 National Academy of Science). **d** *P. aeruginosa* forming surface-attached biofilm (green) and streamers(red) inside curved channel, the latter is able to clog the channel (reproduced with permission from Reference Drescher et al. (2013), copyright 2013 National Academy of Science). **e** *E. coli* swim upstream and super-contaminate narrow ducts (reproduced from Figueroa-Morales et al. (2020), available under the terms of the Creative Commons Attribution Non Commercial License 4.0 (CC BY-NC)

#### Shear-induced retention at surface

Rusconi et al. recorded the concentration profile of bacteria (*B. subtilis*) across the width of a microfluidic channel in a Poiseuille flow (Rusconi et al. (2014b)). As the flow (and shear rate) was increased, they observed a depletion of the central part of the profile of motile cells: up to $70\%$ of cells were depleted from low-shear regions (channel center) and accumulated in high-shear regions (channel boundaries), as shown in Fig. 4a. Non-motile cells, on the other hand, showed a uniform distribution. Combined with mathematical models, they showed that such accumulation results from the competition between the cell alignment with the flow and the stochasticity in the swimming orientation. In contrast to the exclusion that often occurs in colloid transport, the shear-induced “trapping” increases the residence time of bacteria near the surface, thereby facilitating surface adhesion. With a constriction in the flow path, passive particles show no preference for either residence site, but motile bacteria show a preference for both. A counterintuitive observation is the increased cell density after a funnel (Altshuler et al. (2013)). Similarly, motile bacteria in flow also accumulate on the leeward side of cylindrical obstacles (Miño et al. (2018); Lee et al. (2021)). These residential locations become colonization sites afterward (Secchi et al. (2020)).

#### Active motility against flow

At moderate flow rates, motile bacteria may migrate upstream rather than be washed downstream, due to (positive) rheotaxis. Bacterial rheotaxis is a purely physical phenomenon resulting from an interplay of fluid shear, asymmetric body shape, flagellar rotation, and the surface interaction. The shear-induced torque orients the swimming direction of the microorganisms preferentially upstream (Mathijssen et al. (2019)). Figueroa-Morales et al. suggested a link between the rheotaxis and large scale “super-contamination” of biological channels, catheters or water resources (Figueroa-Morales et al. (2020)) (Fig. 4e). Rheotaxis can occur in bulk flow, induced by the chirality and geometrical features of bacteria (Marcos et al. (2012); Jing et al. (2020)). When confined in narrow channels, experiments with artificial microswimmers show that shape asymmetry is no longer necessary (Dey et al. (2022)).

In addition to swimming bacteria, sessile bacteria can also move against the current: In a flow environment, *P. aeruginosa* orient themselves with the type IV pili pole pointing in the opposite direction of the flow, and then they twitch upstream (Siryaporn et al. (2015)) (Fig. 4b). This mechanism allows them to disperse in vasculature-like flow networks. These observations suggest that the dependence of the bacterial orientations on fluid shear, which further influences the transport behavior within porous media.

#### “Catch-bonds” under shear stress

Bacteria have unique feedback mechanisms to counteract biomechanical cues, such as catch-bonds. Thomas et al. reported that the attachment strength of *E. coli* to monomannose-coated surfaces depended on the external shear stress. Increasing the shear stress increased the accumulation of cells on surfaces by up to two orders of magnitude, whereas decreasing the shear stress led to cell detachment (Thomas et al. (2002, 2004, 2008)). Such attachment occurs via “catch-bonds” mediated by the type 1 fimbrial adhesive subunit, FimH, a force-responsive mechanism. At low-shear rates, cells form only the normal “slip-bonds”. At increased shear rates most cells detached, but some stick firmly due to the formation of “catch-bonds” induced by the high shear. Although “catch-bonds” are only reported on the *E. coli* adhesion, FimH is the most common type of bacterial adhesin known, it is possible that other taxa have similar mechanisms.

#### Environmental engineering by microorganisms

Bacteria are able to secret numerous polymeric substances to modify their local environment, as means to protect themselves from harmful environmental conditions and to support the biofilm life form (Flemming and Wuertz (2019)). These substances are generally referred to as extracellular polymeric substances (EPSs), and typical EPS includes polysaccharides, proteins, short-chain DNAs, biosurfactants, and lipids. EPSs tune the surface properties of microbial cells. This could facilitate the transport or retention of the cells depending on the type of the substance (Zhong et al. (2017)). Interestingly, one of the confirmed triggers for EPS production is fluid shear (Weaver et al. (2012); Rodesney et al. (2017)). Prior to adhesion, bacteria produce EPS near the surface. Although still in aqueous solution, EPS forms a weak network through entanglements and ion-mediated intermolecular associations (Ganesan et al. (2016)), which becomes a precursor of biofilm. The Psl exopolysaccharide produced by *P. aeruginosa* not only acts as a “molecular glue” and promotes surface attachment (Ma et al. (2006)), but also acts as an autochemotactic trail that accumulates cells and initiates the colonization (Zhao et al. (2013)).

In saturated, nutrient-rich regions, such as the riverbeds, microorganisms grow in well-developed and highly differentiated biofilms. In addition to the surface-attached, flat biofilms, there is a special type of filamentous biofilm that follows the streamline, called “streamers.” Streamers can cause bioclogging and alter the hydraulic properties of the porous media (Drescher et al. (2013); Battin et al. (2016)) (Fig. 4d).

Even without the production of biochemicals, the swimming motion itself can already modify the environment. Friedlander et al. have reported that when the bacteria swim over a Cassie-Baxter wetted surface (air bubbles trapped in between surface textures), the beating flagella will pump energy into the system, break the Cassie-Baxter wetting state and changing it into a Wenzel state (air bubbles released), thereby increasing the surface area available for adhesion (Friedlander et al. (2013)) (Fig. 4c).

###  Active particles on a random landscape

A Brownian particle in an unconfined space moves diffusely. If the confinement is strong, i.e., when gas molecules diffuse through very narrow pores, the size of which is comparable to or smaller than the mean free path of the gas, their migration is described by Knudsen diffusion, which is one of the models of subdiffusion. Microbial migration within porous media falls into a similar range, where the bacterial “mean free path” (mean run length) is of the same order of magnitude as the typical pore diameter (Duffy et al. (1995)). Note that the “run length” here is not limited to the swimming bacteria, but also refers to the persistent length for twitching bacteria on the surface.

Due to their activity, unconfined bacteria migrate superdiffusively in the short range, and diffusively in the long range. In simulations, they are often represented as active particles, such as active Brownian particles (ABPs) (Zeitz et al. (2017)), run-tumble particles (RTPs) (Reichhardt and Reichhardt (2014)). The overall dynamics depend on several parameters in the system, including the activity of the particles, the reorientation frequency, and the density of obstacles (Chepizhko and Peruani (2013); Reichhardt and Reichhardt (2014); Morin et al. (2017); Zeitz et al. (2017)) Typically, when the density of obstacles is high, and the frequency of reorientation is low (corresponding to long run lengths), the particles will enter a sub-diffusion regime and even become trapped.

Different strategies are required to navigate efficiently in an open space or a disordered environment. Volpe et al have suggested that a diffusive search is more efficient than a ballistic search when navigating a complex topography with boundaries, barriers, and obstacles (Volpe and Volpe (2017)). For the run-tumble particles, several studies using different models have predicted an optimized tumbling probability in order to maximize the diffusivity in disordered environments (Reichhardt and Reichhardt (2014); Licata et al. (2016); Bertrand et al. (2018); Irani et al. (2022)). This is qualitatively explained by experimental observations: increased tumbling frequency also increases the migration efficiency of swimming bacteria inside agarose (Wolfe and Berg (1989)).

More recently, Bhattacharjee et al. have developed a transparent porous medium that allows three-dimensional tracking of bacterial movement within it (Bhattacharjee and Datta (2019a, 2019b); Perez et al. (2021)). They have observed that due to the pore-scale confinement, the bacteria exhibit a “hop-and-trap” motion rather than “run-and-tumble” motion. Cells are intermittently and transiently trapped and then hop to the next position. While “hopping” is determined by pore-scale confinement, and is independent of cellular activity; “trapping” is determined by the competition between pore-scale confinement and cellular activity. Kurzthaler et al have reproduced the “hop-and-trap” behavior in Brownian dynamics simulations of active stiff polymers undergoing run-reverse motion in porous media. They have also proposed a geometric criterion for the optimal spreading when the ballistic “run length” of the bacteria is comparable to the “longest straight path” in the porous medium (Kurzthaler et al. (2021)). Dehkharghani et al identified the key geometric parameter controlling cell transport is the “effective mean free path,” which is calculated as the average distance for randomly sampled, theoretical straight paths between solid surfaces (Dehkharghani et al. (2023)).

**Fig. 5:**
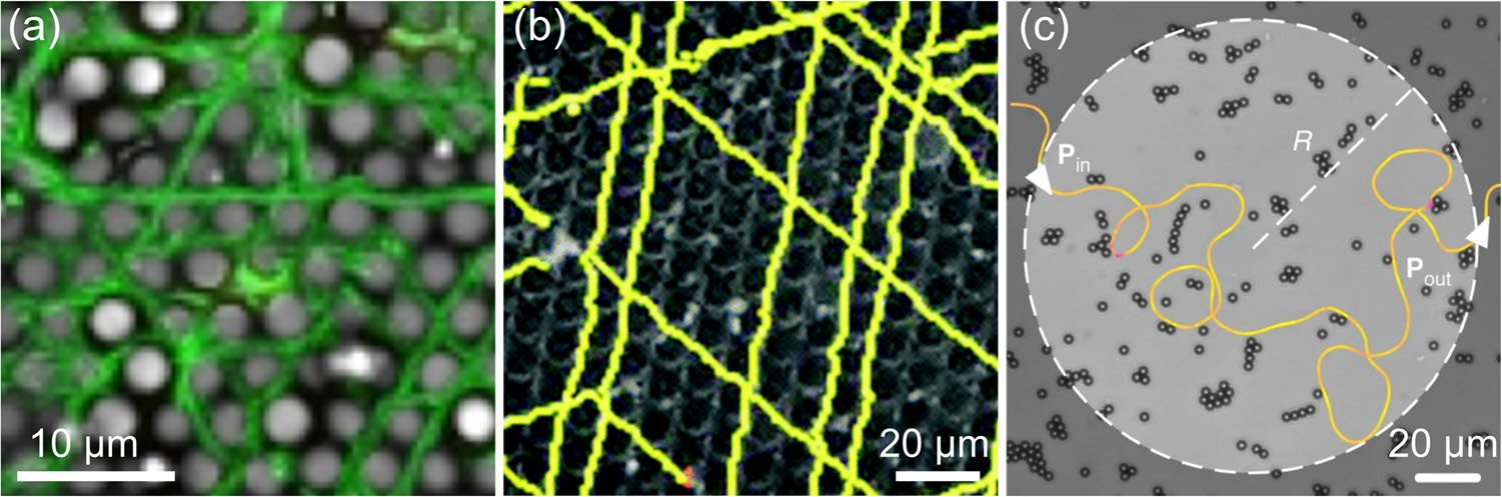
Length scale matching between cells and porous confinements alters the dynamics of microbial locomotion. **a** Twitching *P. aeruginosa* on a surface patterned with arrays of hemispheres following the crystal axis. (reproduced with permission from Reference Chang et al. (2018), copyright 2017 American Chemical Society). **b** Swimming *E. coli* inside a crystal (adapted from Reference Brown et al. (2016), CC BY 3.0). **c** Small obstacles at a certain density can scatter swimming *E. coli* forward (adapted from Reference Makarchuk et al. (2019), CC BY 4.0)

### Microbial motion inside porous media: zooming into the pore scale

Microorganisms are in constant interaction with their local environment while in motion. For example, the hydrodynamic and steric interactions of the swimming bacteria with the pore surface will easily alter their trajectories as discussed in previous sections. Twitching bacteria need to attach their pili to a solid, so the landscape of the environment will also change their dynamics. Figure 5a and b show similar dynamics in two different systems: one is *P. aeruginosa* twitching on a surface patterned with arrays of colloidal hemispheres with an inter-sphere spacing of $4\,\mathrm {\mu m}$ (Chang et al. (2018)), the other is *E. coli* swimming inside a colloidal crystal with the inter-colloid spacing of $\approx 7\,\mathrm {\mu m}$ (Brown et al. (2016)). Both of them show preferential motion along the crystal axes. Note that such induced persistent motion is only observed when the length scale of the bacteria motion and the local environment are matched. When the same *P. aeruginosa* twitches on patterned surfaces with larger or smaller inter-sphere spacing (Chang et al. (2018)), or when swimming bodies with a different rotational dynamics (*E. coli* with shorter flagella and Janus swimmers) swim inside the same colloidal crystal (Brown et al. (2016)), the movement no longer follows the crystal axes and the mean squared displacement (MSD) in the same time interval decreases.

Randomly placed obstacles can also promote the spread of swimming bacteria. Makarchuk et al have reported that small obstacles placed at the right density significantly increase the spread of swimming *E. coli* through individual forward scattering events (Makarchuk et al. (2019)) (Fig. 5c).

### Confined microorganisms: escape or settle down?

Although bacteria are often found in pores comparable to the cell size, it remains to be confirmed whether they have actively chosen to occupy these spaces or have been passively deposited. In addition, the impact of pore size confinement on biological activity and metabolism remains to be elucidated. Existing reports support the hypothesis that bacteria are able to sense the contact with a surface (O’Toole and Wong (2016)), and even discriminate between different surface properties (Friedlander et al. (2013); Yang et al. (2022)). Geng et al reported that mere contact with a surface can reduce the level of cellular respiration in certain strains of *E. coli* (Geng et al. (2014)). One of the mechanisms responsible for surface sensing is the “flagellar dynamometer”: upon contact with a surface, the reduced rotation of the flagellar filament initiates a signalling pathway that triggers a change in bacterial state to swarming or biofilm formation (O’Toole and Wong (2016)). Type IV pili have also been shown to play an important role in surface sensing.

One would naturally expect that geometric confinement would also induce changes in microbial metabolism and dynamics, directly via mechanical, and indirectly via chemical cues. Chu et al demonstrated that the mechanical stress of bacteria filling a microfluidic chamber induces biofilm formation (Chu et al. (2018)). In some other cases, confinement (2 to 3 body sizes) triggers the escape response of bacteria (Lynch et al. (2022)), and acts as a critical cue for *Vibrio fischeri* to colonize their animal host. The acceleration of swimming bacteria under confinement is also seen in other systems (Yin et al. (2022)) and is explained as a hydrodynamic effect. Geometric confinement can also increase the local concentration of chemical signals, inducing quorum sensing that normally occurs only in high cell densities (Carnes et al. (2010)).

Very strict confinement, however, limits the mobility of the bacteria. Experiments show that the limit of flagella-mediated motility is reached when the cell is in a slit of less than $\approx 30\%$ wider than the body width (Männik et al. (2009)). Bacteria (*E. coli* and *B. subtilis* in this study) still have the ability to penetrate such a slit with growth-induced sliding motility (Männik et al. (2009)). Other escape mechanisms exist: *Shewanella putrefaciens* can escape from a narrow pore with a screw-like movement (Kühn et al. (2017)).

## Summary and perspectives

Building on the recent insights gained at the interface of microbial ecology and porous media, in this review article we have focused on key parameters—both physical and biological—which have advanced our understanding of the active processes and feedback governing microbial behavior and physiology under diverse porous settings. How physical constraints, in conjugation with active biophysical mechanisms, mediate microbial fitness and survival is an emerging area of research. Specifically, the ability of microbes to shape their local environments feeds back into their behavior and physiology, triggering interactions at across individual, population, and community scales. More broadly, these new insights shed light on emerging microbiome structures, assemblages, and functions under porous conditions. The active strategies covered in this review should provide the fundamental concepts for dissecting microbial interactions with their neighbors and porous boundaries under ecologically relevant conditions. Taking cues from the nature, systematic mechanistic studies can be designed to uncover the microbial feedback loops and the next generation of bioremediation and health management suites (Borer and Or (2021); Sengupta (2020); Philippot et al. (2023); Jansson et al. (2023); Wheeler et al. (2019); Bansil and Turner (2018)).

As cross-disciplinary multi-scale experimental approaches become the norm, data on microbial behavior and physiology, in relation to the micro-environmental properties, are beginning to be widely accessible. Our ability to zoom into the micro-scale dynamics is expected to reveal how the local environment—both spatially and temporally—shapes the non-equilibrium dynamics underpinning emergent microbial interaction networks. Importantly, new insights into how microbes and microbiomes shape local constraints, have clearly highlighted the need to develop an ensemble approaches to study microbe-matrix interfaces. Machine learning tools including deep neural networks for feature recognition and tracking; and recurrent nets and random forests for time series analysis (Cichos et al. (2020)) could be a timely incorporation to develop a comprehensive understanding. Unravelling microbial interactions and feedback in porous environments will have far-reaching implications for biomedicine, food sciences, sustainability, and environmental biotechnology, alongside fundamental scientific discoveries.

Multiple open questions at the interface of microbes and porous media await exploration. For instance, microbial behavior and interactions in porous environments are modelled as active Brownian particles, taking into account the mean values of key parameters such as cell size, division rates, motility and rotational diffusion. Yet, natural systems are *noisy* in a statistical sense (Dhar et al. (2022); Figueroa-Morales et al. (2020)). Although the role of noise has recently been discussed in the context of biofilm development (Dhar et al. (2022)), its corresponding role in mediating active interactions and feedback under porous constraints remains unexplored. In this context, it would be important to also understand how, and to what extent, strict confinement conditions affect the ability of species to undergo motile-sessile life form shifts. Strict confinement induces secondary physical effects, such as the enhancement of local gradients (chemical, pH, salinity, etc.), suggesting potential roles of confounding factors which could mediate life form shifts in pore-confined microbes. Beyond single microbial species, future studies could be designed to understand the interaction of microbial communities in porous networks (Jansson et al. (2023)). Overlaying such networks with ecologically relevant models of microbial habitats could reveal functional interactions among species, and advance predictive modelling of complex metabolic landscapes with relevant implications for the microbial structures and functions in natural environments (Borer and Or (2021)). Together with bespoke tracking techniques (Rani and Sengupta (2023); Meacock and Durham (2023)), microbial growth and development could be studied systematically over long time scales under controlled porous settings. Finally, porous environments, particularly at nano-scales, can enhance local molecular concentrations and nucleate highly ordered material phases of biological significance (Ulaganathan and Sengupta (2023)), for example, through the selective absorption of microbe-generated lyotropic biosurfactants. The self-assembly of lyotropic liquid crystalline phases may, in turn, impact flows through the porous structures by locally altering the geometry, topology, or surface properties (Sengupta (2013)), ultimately generating biophysical feedback loops within the microbe-inhabited porous environments.

## Data Availability

No datasets were generated or analyzed during the current study.
